# Subacute Reperfusion in Ischemic Hearts: Study of Autophagy and its Possible Interconnection with Receptor-Interacting Protein Kinase 3

**DOI:** 10.31083/j.rcm2306213

**Published:** 2022-06-09

**Authors:** Csaba Horváth, Tanya Ravingerová, M. Saadeh Suleiman, Adriana Adameová

**Affiliations:** ^1^Department of Pharmacology and Toxicology, Faculty of Pharmacy, Comenius University in Bratislava, 83232 Bratislava, Slovak Republic; ^2^Centre of Experimental Medicine, Institute for Heart Research, Slovak Academy of Sciences, 81438 Bratislava, Slovak Republic; ^3^Faculty of Health Sciences, Bristol Heart Institute, The Bristol Medical School, University of Bristol, BS8 Bristol, UK

**Keywords:** myocardial ischemia/reperfusion injury, cell death, autophagy, necroptosis

## Abstract

**Background:**

The role of cardiac autophagy during ischemia and reperfusion (I/R) remains controversial. Furthermore, whether this cell death during I/R is also interconnected with other cell damaging event, such as necroptosis, is insufficiently known. Thus, the aim of this study was to investigate possible links between autophagy and necroptosis in the hearts under conditions of acute I/R injury.

**Methods:**

Langendorff-perfused male Wistar rat hearts were subjected to 30-min global ischemia followed by 10-min reperfusion in the presence of either vehicle or a drug inhibiting the pro-necroptotic receptor-interacting protein kinase 3 (RIP3). Hemodynamic parameters and lactate dehydrogenase (LDH) release were measured to assess heart function and non-specific cell death due to the disruption of plasma membrane.

**Results:**

Immunoblot analysis of left ventricles revealed that early reperfusion suppressed the activation of autophagy as evidenced by the decreased protein expression of Beclin-1, pSer555-ULK1, pSer555-ULK1/ULK1 ratio, and LC3-II/LC3-I ratio. On the other hand, the molecular signalling responsible for autophagy inhibition did not appear to be affected in these I/R settings. RIP3 inhibition during reperfusion significantly mitigated the loss of the plasma membrane integrity but did not improve cardiac function. This pharmacological intervention targeting necroptosis-mediating protein decreased LC3-II expression in I/R hearts, suggesting some effect on autophagosome processing, but it did not significantly alter other signalling pathways involved in autophagy activation or inhibition.

**Conclusions:**

In summary, we showed for the first time that an early reperfusion phase does not promote autophagy and that there may be an interplay between pro-necroptotic protein RIP3 and autophagy with respect to the regulation of autophagosome processing.

## 1. Introduction

Autophagy is generally considered as a process to maintain homeostasis via the removal of damaged cellular organelles as well as malformed and non-functional proteins. However, under certain cardiac pathologic conditions, the advantages of this cellular activity are not as evident [1]. For instance, in the settings of myocardial ischemia/reperfusion (I/R) injury, autophagy plays a different role in the ischemic compared to the reperfusion phase which implicates distinct signalling pathways [2]. Indeed, during ischemia, decreased intracellular ATP production activates AMP-activated protein kinase (AMPK) leading to phosphorylation of the Unc-51-like autophagy activating kinase 1 (ULK1) and inactivation of mammalian target of rapamycin (mTOR) with resultant autophagy initiation to mitigate organ injury and promote cell survival [3]. In contrast, during reperfusion, instead of AMPK, Beclin-1 is activated to promote a higher rate of autophagy with a possible progressive consumption of cellular constituents and finally cell death [4]. In particular, the prolonged phase of reperfusion promotes excessive autophagic program thereby augmenting cell loss. Thus, autophagy can be viewed as a “double-edged sword” in terms of being either cardioprotective or detrimental [1, 5]. Several proteins are involved in autophagy activation (AMPK, pThr172-AMPK, Beclin-1, ULK1, pSer555-ULK1) and its inhibition (Akt, pSer308-Akt, pSer473-Akt, mTOR, pSer2448-mTOR, pSer757-ULK1). They are intertwined in a very complex manner thereby mediating a tight regulation of autophagy [2, 3, 5]. In addition to these autophagic markers, microtubule-associated protein 1A/1B-light chain 3 (LC3) has been recognized as a key marker of the formation and maturation of the double-membrane sequestering vesicles (autophagosomes), and therefore it is considered as a crucial step driving autophagy execution [6, 7, 8].

Necroptosis, a necrosis-like cell death being executed upon the activation of the protein complex consisting of receptor-interacting protein kinase 3 (RIP3) and mixed lineage kinase domain-like pseudokinase (MLKL) [9, 10], has been shown to underlie cardiac I/R damage [11, 12, 13]. A limited number of studies have also provided evidence indicating a linkage of necroptotic cell death with the autophagic process. In fact, necroptosis has been suggested to act as an upstream inhibitor of autophagy [14] while others have suggested that over-stimulated autophagy initiates necroptotic cell loss thereby placing necroptosis as a downstream event [15]. It is worth mentioning that many of the studies dealing with a plausible interplay between autophagy and necroptosis have used chronic models of I/R employing a long duration of reperfusion (from several minutes to several days). In our recent study, we have reported that a short reperfusion phase is unlikely to be responsible for necroptotic cell death due to I/R despite some evidence suggesting loss of cellular integrity, and mitochondrial swelling [16]. Considering these facts and possible underlying mechanisms of such cellular damage under such shorter conditions of reperfusion, we analysed the signalling pathways involved in both autophagy activation and inhibition. In addition, RIP3 inhibition was used to assess a possible interplay between autophagy and necroptosis signalling, mainly a non-canonical signalling being associated with mitochondria. Understanding the involvement and interaction between necroptosis and autophagy early after reperfusion might help to identify targets for early therapeutic intervention.

## 2. Methods

### 2.1 Experimental Protocol and Assessment of Mechanic Heart Function

Detailed study protocol has been described previously [16]. In brief, adult male Wistar rats, supplied by Charles River Laboratories (Oxford, UK), were randomly divided into the groups: perfusion only (Control; n = 6); control + RIP3 inhibitor—GSK’872 (n = 6); I/R (n = 7); I/R + GSK’872 (n = 7). Intraperitoneal injection of sodium pentobarbital (60 mg/kg) was used to induce anaesthesia. Hearts were retrogradely perfused with a modified Krebs-Henseleit buffer (pH = 7.4; 95% $O_{2}$ and 5% ${\mathrm{CO}}_{2}$) in a Langendorff mode at a constant perfusion pressure (73 mmHg) and temperature (37.5 ± 0.2 °C) as described previously [16]. After stabilization period, during which measured functional parameters of the hear were constant, a 30-minute global ischaemia was induced followed by a 10-min reperfusion. This duration of reperfusion was chosen based on our hypothesis that RIP3 due to affecting the downstream signalling molecules may modulate mitochondrial processes, in particular mitochondrial swelling, which in our hands was most evident upon 10 minutes of reoxygenation [16]. Hearts were perfused with either vehicle (0.004% v/v dimethylsulfoxide (DMSO); Control and I/R group) or GSK’872 (250 nmol/L) dissolved in DMSO stock given during a 10-min perfusion/reperfusion period. The rationale for the choice of GSK’872 dose was based on its IC50 [17]. PowerLab/8SP Chart 7 software (ADInstruments, Inc., Castle Hill, Australia) was used to measure mechanical function of the heart. Left ventricular developed pressure (LVDP) is expressed as percentage of baseline (end of stabilization) values. After the end of the protocol, the hearts were frozen in liquid nitrogen and stored at –80 °C until further processing.

### 2.2 Determination of Lactate Dehydrogenase Release

Lactate dehydrogenase (LDH) activity was determined by using a modified protocol of Bergmeyer H–U 1963 in the effluent perfusate collected from the hearts during the whole 10-minute reperfusion. Briefly, 80 μL of the sample was mixed with 910 μL of the buffer (pH 7.4) containing 100 mmol/L triethanolamine and 100 μmol/L reduced beta-nicotinamide adenine dinucleotide (β-NADH). The change of absorbance at 340 nm (A340) was recorded using a spectrophotometer over 5 min at 37 °C immediately after addition of 10 μL of 0.1 mol/L sodium pyruvate to the reaction mixture.

### 2.3 SDS-PAGE and Immunoblotting

Left ventricular tissue samples of the hearts were processed for immunoblot analysis by sodium dodecyl sulfate-polyacrylamide gel electrophoresis (SDS-PAGE) and Western blotting as described previously [16]. Briefly, post-electrophoresis, proteins were transferred onto polyvinylidene fluoride (PVDF) membranes (Immobilon-P, Merck Millipore) and incubated with primary antibodies against HMGB1 (#6893, Cell Signaling Technology, Danvers, MA, USA), pan-Akt (#4691, Cell Signaling Technology, Danvers, MA, USA), pSer473-Akt (#4060, Cell Signaling Technology, Danvers, MA, USA), pThr308-Akt (#4056, Cell Signaling Technology, Danvers, MA, USA), AMPK (A3730, Sigma-Aldrich, Darmstadt, Germany), pThr172-AMPK (#2531, Cell Signaling Technology, Danvers, MA, USA), mTOR (#2983, Cell Signaling Technology, Danvers, MA, USA), pSer2448-mTOR (ab109268, Abcam, Cambridge, UK), ULK1 (#8054, Cell Signaling Technology, Danvers, MA, USA), pSer757-ULK1 (#14202, Cell Signaling Technology, Danvers, MA, USA), pSer555-ULK1 (#5869, Cell Signaling technology, Danvers, MA, USA), LC3A/B (#12741, Cell Signaling Technology, Danvers, MA, USA), Beclin-1 (ab32064, Abcam, Cambridge, UK). Subsequently, membranes were incubated with horseradish peroxidase (HRP)-conjugated secondary donkey anti-rabbit immunoglobulin G (IgG) antibody (711-035-152, Jackson ImmunoResearch, West Grove, PA, USA). Signals were detected using enhanced chemiluminescence (Crescendo Luminata, Merck Millipore, Burlington, MA, USA) and captured by a chemiluminescence imaging system (myECL imager, Thermo Scientific, Waltham, MA, USA). Total protein staining of membranes with Ponceau S assessed by scanning densitometry was used as the loading control in total tissue lysates [18]. Relative expression of protein bands of interest was calculated by normalizing the intensity of a protein band to its whole lane protein staining intensity.

### 2.4 Statistical Analysis

Statistical analysis complies with the recommendations on study design and analysis in experimental pharmacology [19]. Data are expressed as means ± standard error of means (SEM) for the number of animals in the group. Mixed-model ANOVA (MMA) was used to compare time-course LDH release of perfusion-only and I/R groups. Two-way ANOVA (2WA) and Holm–Sidak’s post hoc tests were applied for comparison of differences in variables with normal distribution between the 4 groups (“Early reperfusion” factor—presence of ischemia/reperfusion; “GSK’872” factor—presence of RIP3 inhibitor; “Early reperfusion x GSK’872” factor—the interaction of the two factors). GraphPad Prism 9.00 for Windows (GraphPad Software, San Diego, CA, USA) was used for analyses. Differences between groups were considered significant when *p *< 0.05.

## 3. Results

**Fig. 1.**
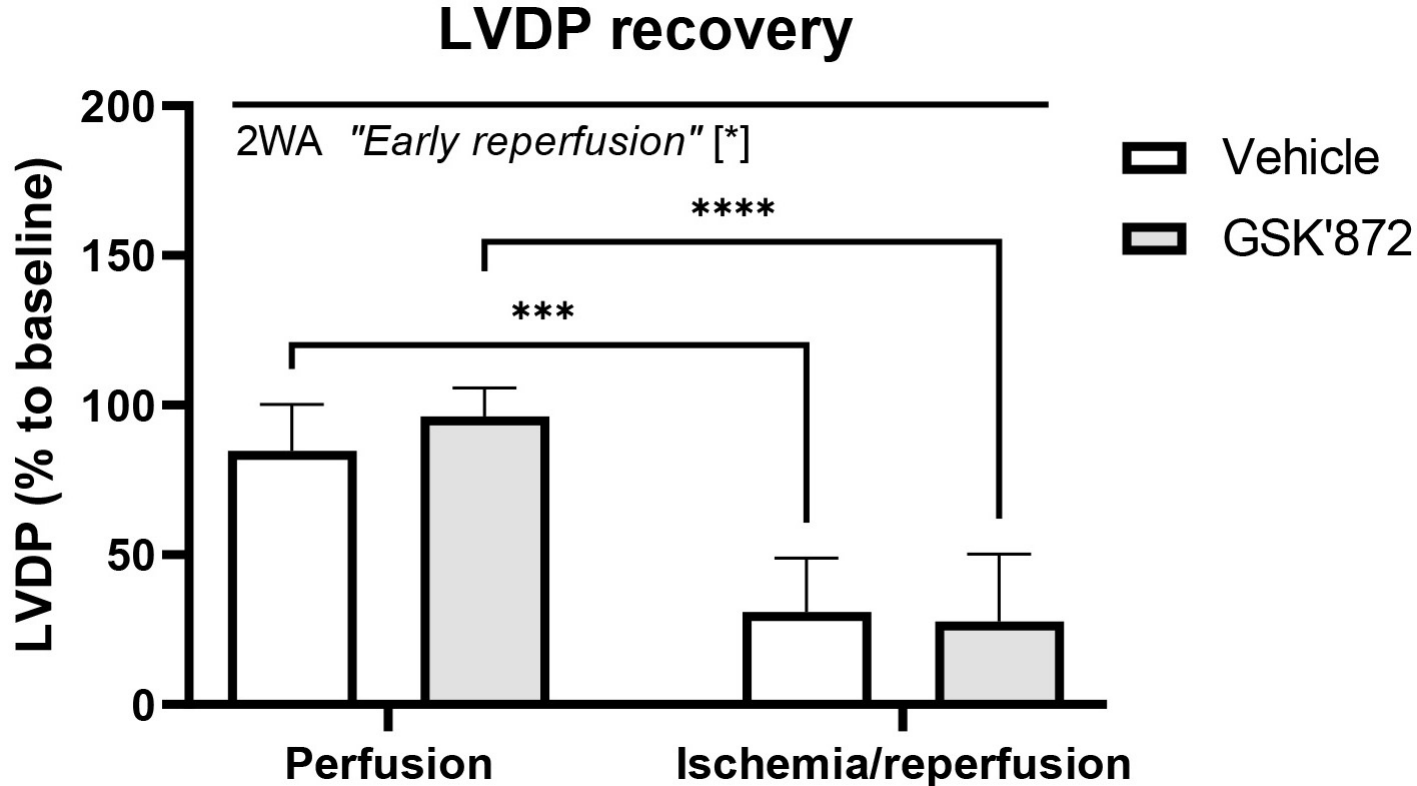
**Recovery of left ventricular developed pressure (LVDP) in the hearts subjected to perfusion-only or ischemia/reperfusion in the presence/absence of RIP3 inhibition**. Data are presented as mean ± SEM; * *p *< 0.05. 2WA—two-way ANOVA; “Early reperfusion” factor—presence of ischemia/reperfusion.

Left ventricular pressure recordings revealed that contractile function of the heart was significantly impaired due to ischemia followed by 10-min reperfusion and that the selective inhibitor of the necroptosis-mediating protein RIP3 did not abrogate myocardial dysfunction. Indeed, post-ischemic recovery of LVDP was comparable in both non-treated and GSK’872-treated I/R group (Fig. 1).

**Fig. 2.**
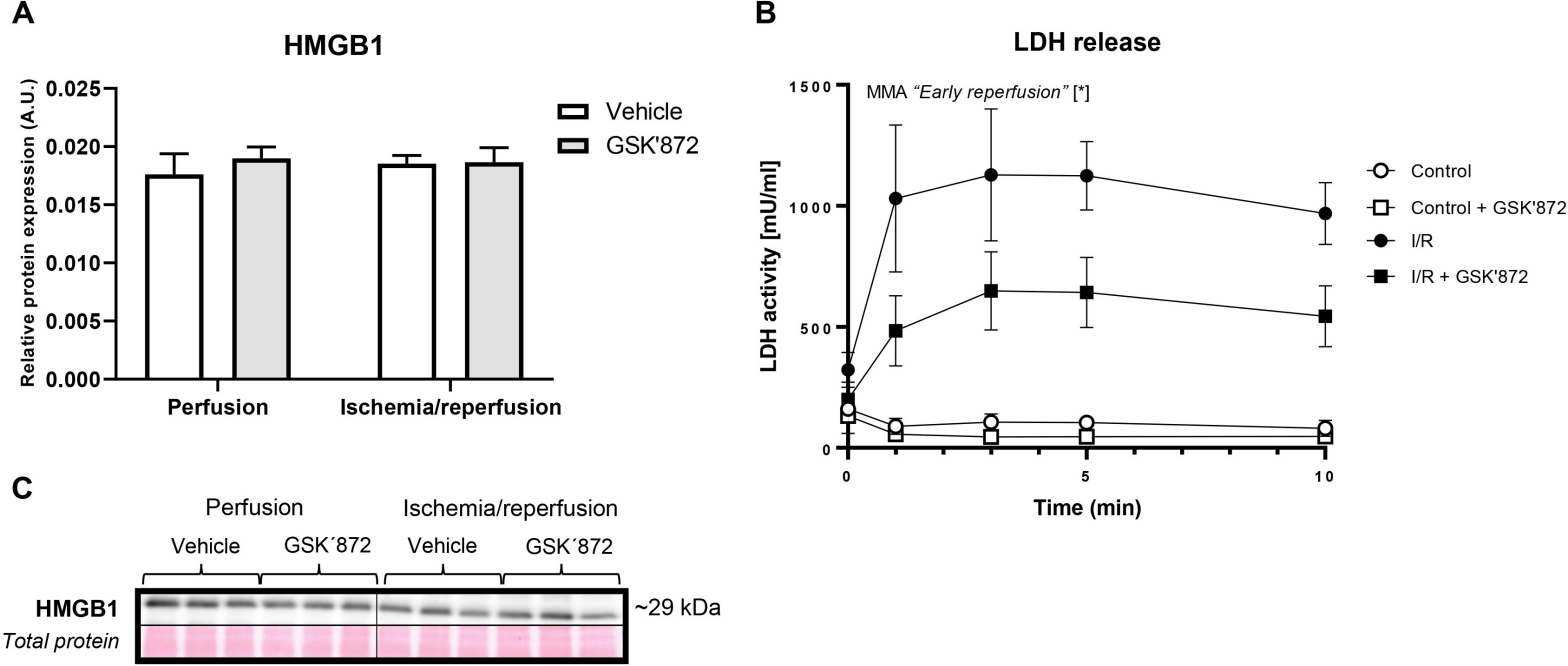
**Evaluation of markers indicating cell disruption in the left ventricle of rat hearts**. (A) Immunoblot quantification of HMGB1. (B) LDH release. (C) Representative immunoblot and total protein staining. Data are presented as mean ± SEM; * *p *< 0.05. MMA—mixed-model ANOVA; “Early reperfusion” factor—presence of ischemia/reperfusion.

There was no difference in the myocardial expression of high mobility group box 1 protein (HMGB1) in response to I/R damage (Fig. 2A,C). A significant release of LDH was seen during reperfusion and this release was significantly reduced in the presence of RIP3 inhibitor indicating the relative maintenance of the plasma membrane integrity (Fig. 2B).

**Fig. 3.**
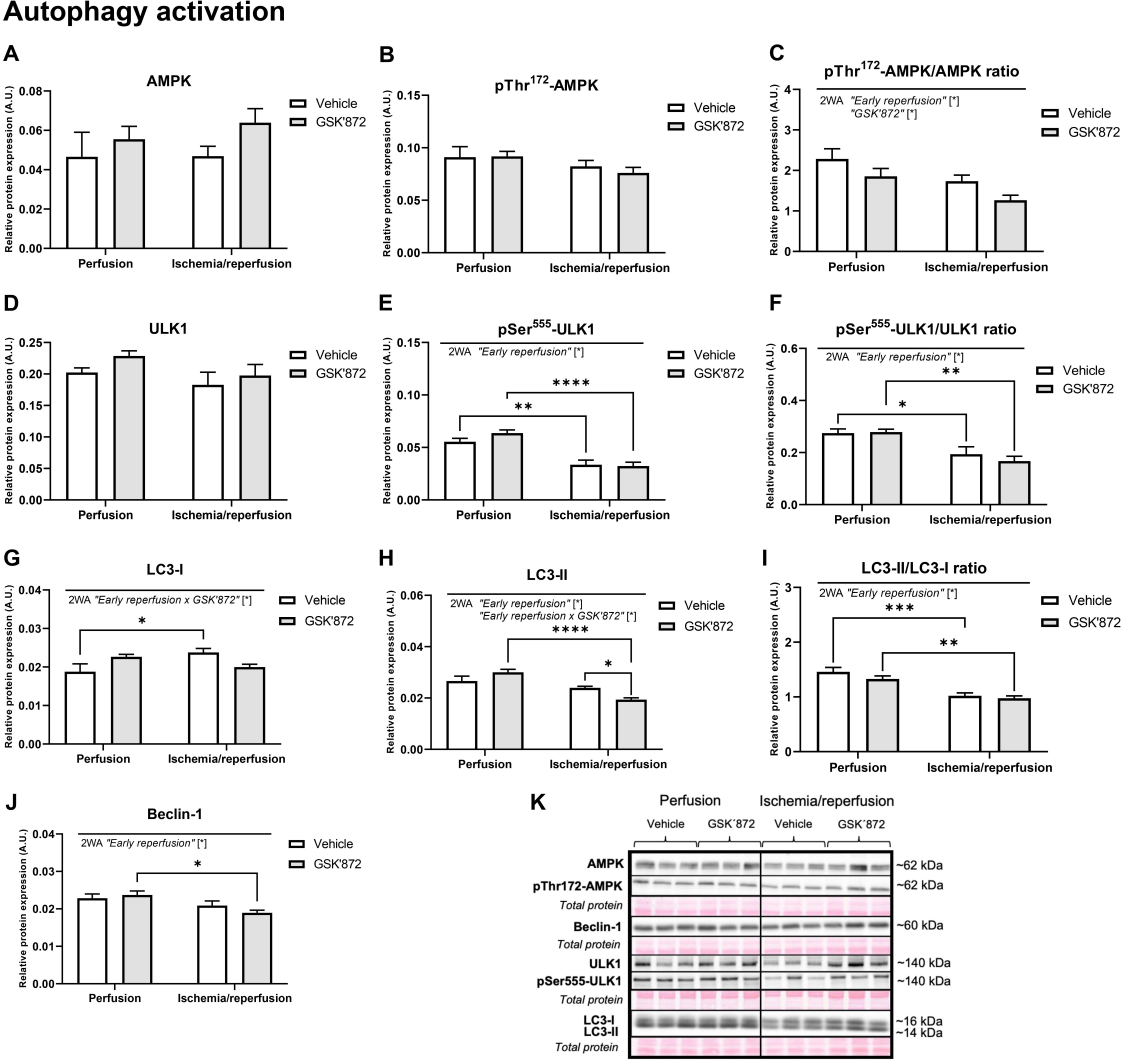
**Analysis of activation of autophagic signalling in the left ventricle of rat hearts**. (A–J) Immunoblot quantification of AMPK, pThr172-AMPK, pThr172-AMPK/AMPK ratio, ULK1, pSer555-ULK1, pSer555-ULK1/ULK1 ratio, LC3-I, LC3-II, LC3-II/LC3-I ratio, Beclin-1. (K) Representative immunoblots and total protein staining. Data are presented as mean ± SEM; * *p *< 0.05. 2WA—two-way ANOVA; “Early reperfusion” factor—presence of ischemia/reperfusion, “GSK’872” factor—presence of RIP3 inhibitor; “Early reperfusion x GSK’872” factor—interaction of the two factors.

**Fig. 4.**
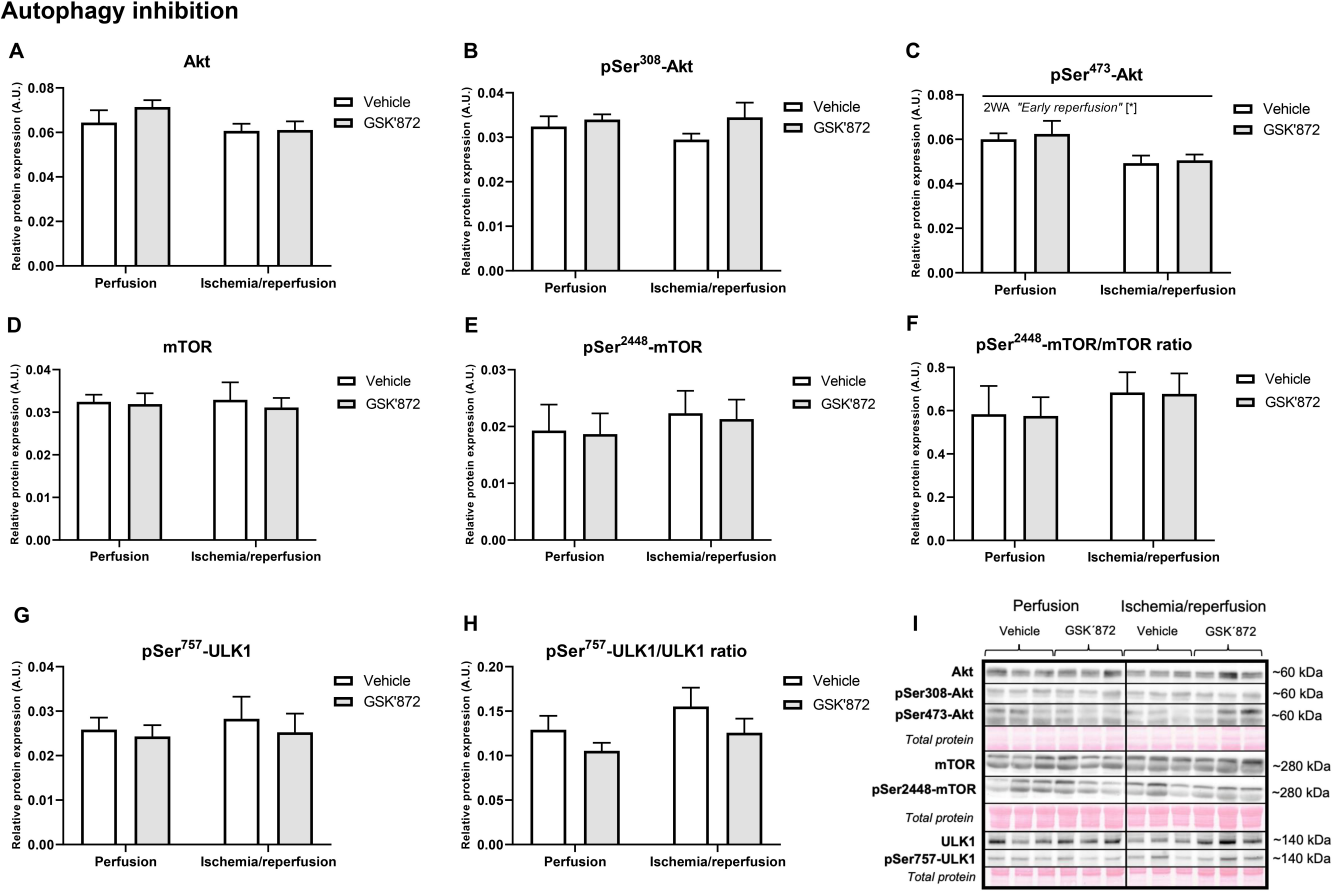
**Analysis of inhibition of autophagic signalling in the left ventricle of rat hearts**. (A–H) Immunoblot quantification of Akt, pThr308-Akt, pSer473-Akt, mTOR, pSer2448-mTOR, pSer2448-mTOR/mTOR ratio, pSer757-ULK1, pSer757-ULK1/ULK1 ratio. (I) Representative immunoblots and total protein staining. Data are presented as mean ± SEM; * *p *< 0.05. 2WA—two-way ANOVA; “Early reperfusion” factor—presence of ischemia/reperfusion.

The levels of the main proteins regulating autophagy are shown in Figs. 3,4. The expression of both total (Fig. 3A) and phosphorylated AMPK at Thr172 (Fig. 3B), which is known to be active during ischemia rather than in reperfusion [2], was unaltered in I/R hearts independently of RIP3 inhibition. On the other hand, the pThr172-AMPK/AMPK ratio was suppressed by both the I/R intervention and RIP3 inhibition (Fig. 3C). The pattern of levels of the total ULK1 was consistent with the expression of AMPK (Fig. 3D). On the other hand, after 10-min of reperfusion and regardless of the presence or absence of GSK’872 there was a decrease in the active phosphorylated (Ser555) form of ULK1 (Fig. 3E) as well as its ratio to total ULK1 (Fig. 3F). The expression of LC3-I having a conspicuous role in the initiation of autophagosome formation [7], was increased due to such an acute I/R treatment, while two-way ANOVA analysis revealed the significance in the interaction of I/R and the treatment only (Fig. 3G). In contrast, LC3-II, which translocates rapidly to nascent autophagosomes [7], was decreased by I/R. Moreover, RIP3 inhibition under I/R caused even more prominent suppression in LC3-II expression (Fig. 3H). The conversion of LC3-I to LC3-II, which is indicative of autophagic activity [8], was suppressed by I/R irrespective of the presence or absence of the anti-necroptotic agent (Fig. 3I). Nevertheless, at this early reperfusion phase of previously ischemic heart the expression of Beclin-1 was suppressed (Fig. 3J) whilst the presence of GSK’872 did not affect this autophagic mechanism. Representative immunoblots of the investigated proteins are shown in Fig. 3K. During early reperfusion, except for pThr473-Akt being downregulated, none of the investigated proteins involved in autophagy inhibition were altered. RIP3 inhibition was unable to modify this part of autophagic signalling (Fig. 4A–I).

## 4. Discussion

In this study we showed for the first time that a brief 10-min reperfusion of previously ischemic hearts suppressed autophagy activation and did not affect the molecular signalling responsible for autophagy inhibition. We also found that RIP3 inhibition was associated with downregulation of LC3-II expression in the I/R hearts, indicating a possible role for RIP3 in the processing of the autophagosome. In addition, RIP3 inhibition reduced the I/R—mediated disruption of the plasma membrane. Surprisingly, this cardioprotective effect was not translated into an improvement in functional recovery.

Great effort has been made to understand the role of autophagy in the pathophysiology of myocardial I/R injury. Currently, it is accepted that sub-chronic ischemia is sufficient to activate autophagic flux, possibly due to the stimulation of AMPK mainly via phosphorylation on Thr172 [2, 3, 20]. These changes are viewed as an adaptive mechanism to degrade damaged organelles and proteins to finally mitigate ischemic damage [2]. During reperfusion, however, the nature of autophagy seems to be more detrimental. Despite being active during ischemia, decreased activity of AMPK during reperfusion was associated with the aggravation of myocardial injury [21]. In contrast, a pharmacological approach mediating its sustained activity under such pathologic conditions has been shown to have cardioprotective effects [22]. In our hands, the levels of total and phosphorylated forms of AMPK did not show significant changes during I/R with or without treatment with RIP3 inhibitor. However, there was a fall in the phosphorylation of AMPK at pThr172, when expressed as pThr172-AMPK/AMPK ratio, as a consequence of the intervention and treatment. In addition, these hearts subjected to 30-min ischemia followed by 10-min reperfusion exhibited a decrease in the expression of Beclin-1. These findings are in contrast with those reported in a study employing a model of 30-min ischemia followed by 30-min reperfusion, in which the expression of Beclin-1 was upregulated indicating an increased rate of autophagy initiation [2]. Indeed, this suggests a dynamic process of Beclin-1 activity depending on the extent of I/R injury and subsequent cell death over the duration of the reperfusion phase. It is likely that the autophagosome processing documented as the LC3-II/LC3-I ratio was suppressed by early reperfusion irrespective of the presence or absence of the RIP3 inhibitor. Collectively, these findings suggest that 10-min reperfusion is insufficient to stimulate autophagy and rather dampens its induction without affecting the execution of this catabolic process. Thus, it can be hypothesized that such a short reperfusion phase does not mitigate organ injury and promote cell survival, and at the same time, it does not promote the degradation of cellular organelles either. In contrast, there is evidence that a longer duration of reperfusion facilitates the overactivation of autophagy eliciting detrimental effects [2, 4]. In this regard, it can be mentioned that overactivated autophagy due to a longer reperfusion phase might turn into autophagy-like cell death and/or can interfere with necroptosis [1, 4, 5, 14, 15]. The activation of this form of programmed necrosis via the canonical RIP3–MLKL pathway has been documented after 40-min of reperfusion, but not after 10-min reperfusion [16], and has been associated with impaired heart function [13]. In post-myocardial infarction heart failure, representing another model of a chronic reperfusion phase, both necroptosis and autophagy seem to be interweaved in a complex manner and therefore, the potential autophagy–necroptosis linkage might underlie the specific phenotypes of such syndrome [14, 15, 23]. In patients with end-stage heart failure, autophagy might act as an upstream activator of necroptosis [15]. On the other hand, another study pointed out that in post-ischemic cardiomyopathy, necroptosis precedes autophagy and serves rather as an upstream inhibitor of autophagic flux [14]. To the best of our knowledge, this is the first study examining a relationship between autophagy and necroptosis, and assessed by pharmacological targeting of RIP3, in an early phase of reperfusion. By using two-way ANOVA analysis, we identified an interactive effect of I/R intervention and treatment on the expression of the autophagosome markers LC3-I and LC3-II, thereby suggesting a potential mechanism of the modulation of autophagy execution due to RIP3 inhibition in the presence of I/R. Because interaction between the autophagosome and the necrosome, an amyloid-like pro-necroptotic protein complex [9, 10], has been documented [24] such RIP3-mediated changes in both LC3-I and LC3-II under conditions of I/R may suggest a mechanism how the pro-necroptotic protein RIP3 might affect autophagy. On the other hand, it should be mentioned that other autophagic markers involved in either autophagy activation or inhibition were not altered by RIP3 inhibition in I/R hearts.

## 5. Conclusions

In summary, we showed for the first time that brief reperfusion of previously ischemic hearts was able to dampen the activation of autophagy and had no influence on the molecular signalling involved in autophagy inhibition, despite a significant impairment of the heart function. It is also likely that in such subacute reperfusion phase, there might be an interplay between pro-necroptotic protein RIP3 and autophagy with respect to the regulation of autophagosome processing. The pharmacological targeting of RIP3 prevented the loss of the plasma membrane integrity but was unable to mitigate the heart dysfunction. More detailed analyses are needed to prove a concept we proposed in this study and describe a plausibly more complex action of RIP3 in this regard.
