# Correction: Horváth et al. Subacute Reperfusion in Ischemic Hearts: Study of Autophagy and Its Possible Interconnection With Receptor-Interacting Protein Kinase 3. Reviews in Cardiovascular Medicine. 2022; 23(6): 213

**DOI:** 10.31083/RCM58067

**Published:** 2026-09-16

**Authors:** Csaba Horváth, Tanya Ravingerová, M. Saadeh Suleiman, Adriana Adameová

**Affiliations:** ^1^Department of Pharmacology and Toxicology, Faculty of Pharmacy, Comenius University in Bratislava, 83232 Bratislava, Slovak Republic; ^2^Centre of Experimental Medicine, Institute for Heart Research, Slovak Academy of Sciences, 81438 Bratislava, Slovak Republic; ^3^Faculty of Health Sciences, Bristol Heart Institute, The Bristol Medical School, University of Bristol, BS8 1UD Bristol, UK

The authors would like to correct Fig. 3 in the article entitled “Subacute Reperfusion in Ischemic Hearts: Study of Autophagy and its Possible Interconnection with Receptor-Interacting Protein Kinase 3” [1]. During figure assembly, an incorrect image was inadvertently used for the AMP-activated protein kinase (AMPK) immunoblot bands in panel K. The corrected panel K now presents the appropriate AMPK bands obtained from the original experiment. This correction is limited to the AMPK immunoblot image. The authors confirm that this correction does not affect the results or conclusions of this paper. The quantitative data and statistical analyses presented in Fig. 3A–J were generated from the correct original data and remain unchanged.

The authors sincerely apologize for this error and any inconvenience it may have caused. The authors have provided a corrected version of Fig. 3 here:

**The original Fig. 3:**

**Fig. 3.**
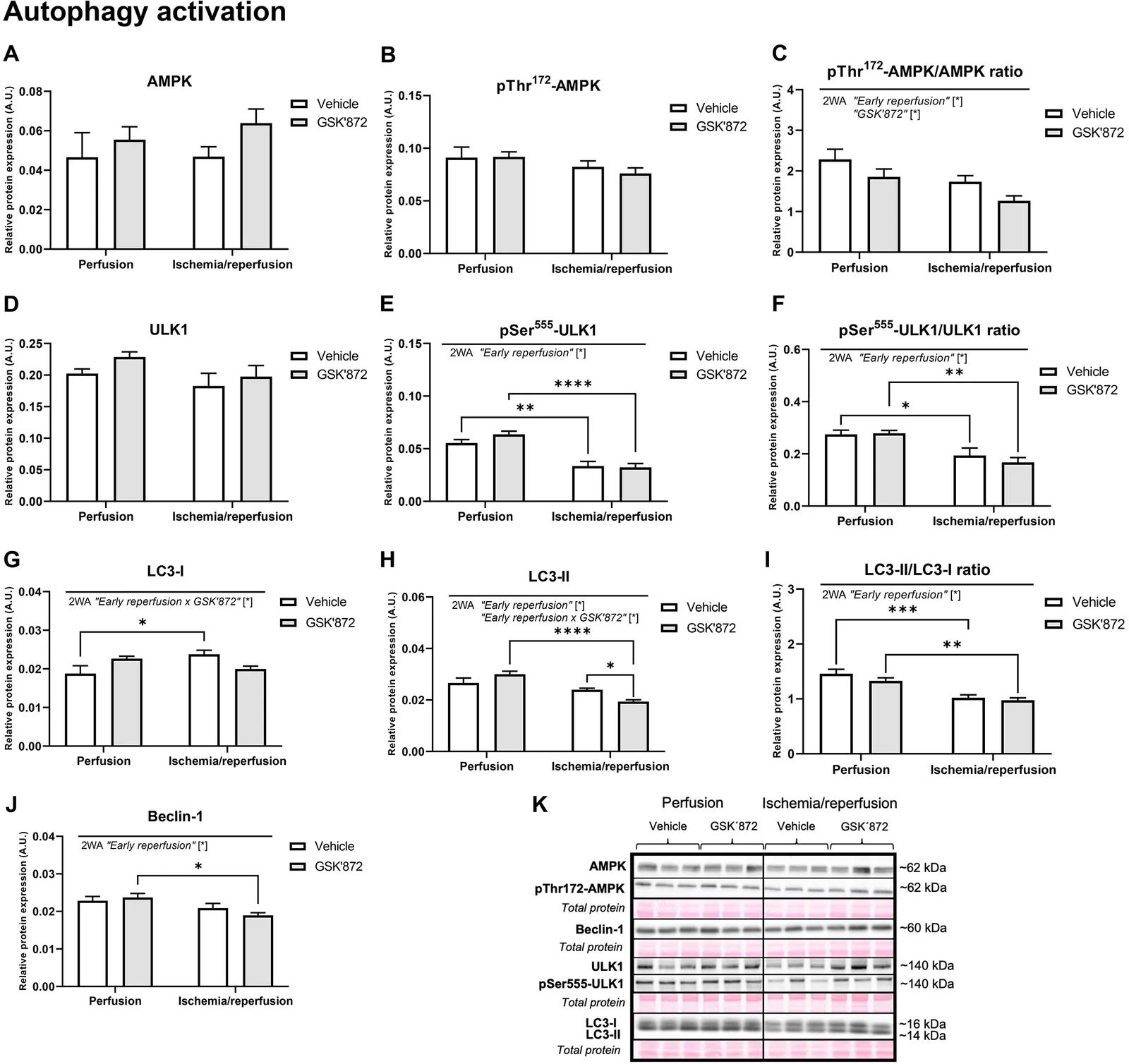
**Analysis of activation of autophagic signalling in the left ventricle of rat hearts. **(A–J) Immunoblot quantification of AMPK, pThr172-AMPK, pThr172-AMPK/AMPK ratio, ULK1, pSer555-ULK1, pSer555-ULK1/ULK1 ratio, LC3-I, LC3-II, LC3-II/LC3-I ratio, Beclin-1. (K) Representative immunoblots and total protein staining. Data are presented as mean ± SEM; * *p* < 0.05; ** *p* < 0.01; *** *p* < 0.001; **** *p* < 0.0001. 2WA—two-way ANOVA; “Early reperfusion” factor—presence of ischemia/reperfusion, “GSK’872” factor—presence of RIP3 inhibitor; “Early reperfusion x GSK’872” factor—interaction of the two factors. AMPK, AMP-activated protein kinase; ULK1, Unc-51-like autophagy activating kinase 1; LC3, light chain 3.

**The corrected Fig. 3:**

**Fig. 3.**
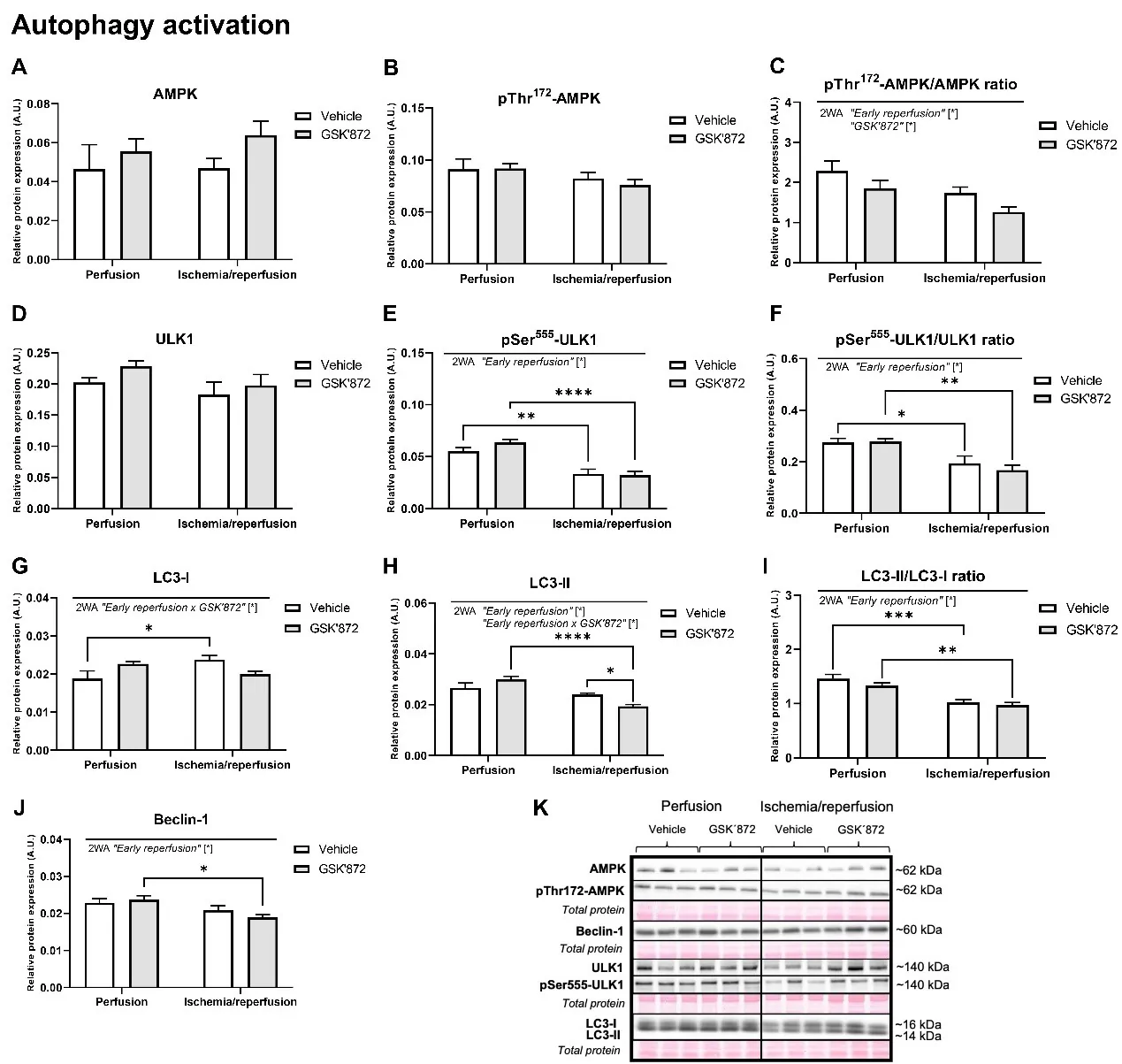
**Analysis of activation of autophagic signalling in the left ventricle of rat hearts. **(A–J) Immunoblot quantification of AMPK, pThr172-AMPK, pThr172-AMPK/AMPK ratio, ULK1, pSer555-ULK1, pSer555-ULK1/ULK1 ratio, LC3-I, LC3-II, LC3-II/LC3-I ratio, Beclin-1. (K) Representative immunoblots and total protein staining. Data are presented as mean ± SEM; * *p* < 0.05; ** *p* < 0.01; *** *p* < 0.001; **** *p* < 0.0001. 2WA—two-way ANOVA; “Early reperfusion” factor—presence of ischemia/reperfusion, “GSK’872” factor—presence of RIP3 inhibitor; “Early reperfusion x GSK’872” factor—interaction of the two factors. AMPK, AMP-activated protein kinase; ULK1, Unc-51-like autophagy activating kinase 1; LC3, light chain 3.

This has been corrected as of 23 July 2026. The authors apologize for this error.
